# Clinical characteristics and overall survival nomogram of second primary malignancies after prostate cancer, a SEER population-based study

**DOI:** 10.1038/s41598-020-80534-4

**Published:** 2021-01-14

**Authors:** Yi Liu, Peipei Zhang, Yinghao Zhang, Lichuan Zheng, Wenbo Xu, Dongtao Hou, Zhengjun Kang

**Affiliations:** 1 Department of Urology, The Fifth Affiliated Hospital of Zhengzhou University, Zhengzhou University, Kangfu Street, Zhengzhou, 450052 Henan China; 2 Department of Pediatrics, The Fifth Affiliated Hospital of Zhengzhou University, Zhengzhou University, Zhengzhou, Henan China; 3 Department of Urology, Xinzheng Hospital, Zhengzhou, Henan China

**Keywords:** Risk factors, Urology, Cancer, Urological cancer, Prostate cancer

## Abstract

Prostate cancer (PCa) is the most prevalent cancer among males and the survival period of PCa has been significantly extended. However, the probability of suffering from second primary malignancies (SPMs) has also increased. Therefore, we downloaded SPM samples from the SEER database and then retrospectively analyzed the general characteristics of 34,891 PCa patients diagnosed between 2000 and 2016. After excluding cases with unknown clinical information, 2203 patients were used to construct and validate the overall survival (OS) nomogram of SPM patients after PCa. We found that approximately 3.69% of PCa patients were subsequently diagnosed with SPMs. In addition, the three most prevalent sites of SPM were respiratory and intrathoracic organs, skin, and hematopoietic system. The top three histological types of SPMs were squamous cell carcinoma, adenoma and adenocarcinoma, nevi and melanoma. Through univariate and multivariate Cox regression analysis, we found that the site of SPM, age, TNM stage, SPM surgery history, and PCa stage were associated with the OS of SPM. By virtue of these factors, we constructed a nomogram to predict the OS of SPM. The C-index in the training set and validation set were 0.824 (95CI, 0.806–0.842) and 0.862 (95CI, 0.840–0.884), respectively. Furthermore, we plotted the receiver operating characteristic curve (ROC) and the area under curve (AUC) which showed that our model performed well in assessing the 3-year (0.861 and 0.887) and 5-year (0.837 and 0.842) OS of SPMs in the training and validation set. In summary, we investigated the general characteristics of SPMs and constructed a nomogram to predict the prognosis of SPM following PCa.

## Introduction

Prostate cancer (PCa) is the most prevalent cancer among men, and it is estimated that 3.6 million men in the United States have a history of PCa in 2019^1^. In addition, in 2020, approximately 191,930 new PCa cases have been registered and 33,330 people have died of PCa in the United States in 2020^2^. Owing to prostate‐specific antigen (PSA) screening, digital rectal examination (DRE), and transrectal ultrasound (TRUS) followed by ultrasound-guided biopsy, PCa can be diagnosed at its early stage. Treatment options for PCa, such as, prostatectomy, androgen deprivation therapy (ADT), chemotherapy, and radiotherapy (RT) have also greatly improved the survival rate of PCa^3–5^. Due to early diagnosis and treatment, the 5‐year relative survival rate of PCa has increased to 98%. Moreover, the death rate of PCa has dropped by 52% from its peak^2^.

Despite the extended survival period of cancer, some people may suffer from the second primary malignancies (SPMs)^6,7^. Previous research demonstrated that about 11.3% of PCa patients were diagnosed with SPMs^8^. According to two large-scale studies in Sweden and Germany, the most frequently detected SPMs originated from PCa patients, accounting for 22.5% and 16.9% of all SPMs, respectively^9^. However, mechanisms of triggering conversion to SPMs are unclear, resulting in diagnostic uncertainty and delays in the diagnosis and treatment of SPMs. The underlying causes of SPMs may include environmental and lifestyle-related factors (e.g., smoking)^10^, genetic factors^11^ and treatment-related exposures (e.g., radiotherapy (RT))^12,13^. Although the mechanism of SPMs is vague, the survival period of patients will be shortened once they are diagnosed with SPMs, and a former study has proved that adolescents and young adults with SPMs have worse survival than those with only primary cancer^14^.

Nomogram created by regression analysis has been widely employed to predict the prognosis of diverse cancers^15^ because of its simplicity, intuitiveness, and practicality. It has been used for bladder cancer^16^, cervical cancer^17^, primary gliosarcoma^18^, and many other diseases. The efficiency of nomogram has been proved and has even become a new standard.

We have realized that it is of great significance for treatment providers and PCa survivors to understand the incidence and prognosis of SPMs after PCa. Therefore, in this study, we aimed to investigate the general characteristics of SPMs and construct a nomogram to predict the 3-year and 5-year survival of SPMs following PCa.

## Materials and methods

### Data source and study design

We extracted SPM cases from 18 population-based registries (2000–2016) in the Surveillance, Epidemiology, and End Results (SEER) database using SEER* Stat version 8.3.6. Clinicopathological data of interest were extracted, including age, race, TNM stage, site of SPM, histological type of SPM and PCa, surgery history of SPM and PCa, marital status, follow-up time, and latency time between PCa and SPM. To make our results more accurate, we adopted the Warren criterion to identify SPM. SPMs were identified as cancers histologically different from the initial primary cancer (IPC), with a latency period of not less than 6 months to exclude errors caused by metastasis and recurrence^19^.

First, we downloaded a total of 68,954 PCa cases from the SEER database. The inclusion criteria were as follows: 1. diagnosed age greater than 18 years; 2. A record of malignant behavior; 3. patients with complete survival data and follow-up information. The exclusion criteria were as below: 1. latency period between IPC and SPM shorter than 6 months; 2. patients with only autopsy or death certificate records. Then, after excluding 33,702 patients with the same histology as PCa, there remained 34,891 patients diagnosed with SPM. Patients with unknown information were also excluded, including no TNM stage: n = 24,452, unknown history of surgery: n = 184, unknown marital status: n = 121, unknown lymph node removed (LNR): n = 10, and no stage of PCa: n = 7921. Ultimately, we identified 2203 qualified cases, which were then divided into the training set (n = 1543) and the validation set (n = 660). The training set was used to identify prognostic factors and built a nomogram based on these factors. The training set and validation set were used for internal and external validation, respectively.

**Figure 1:**
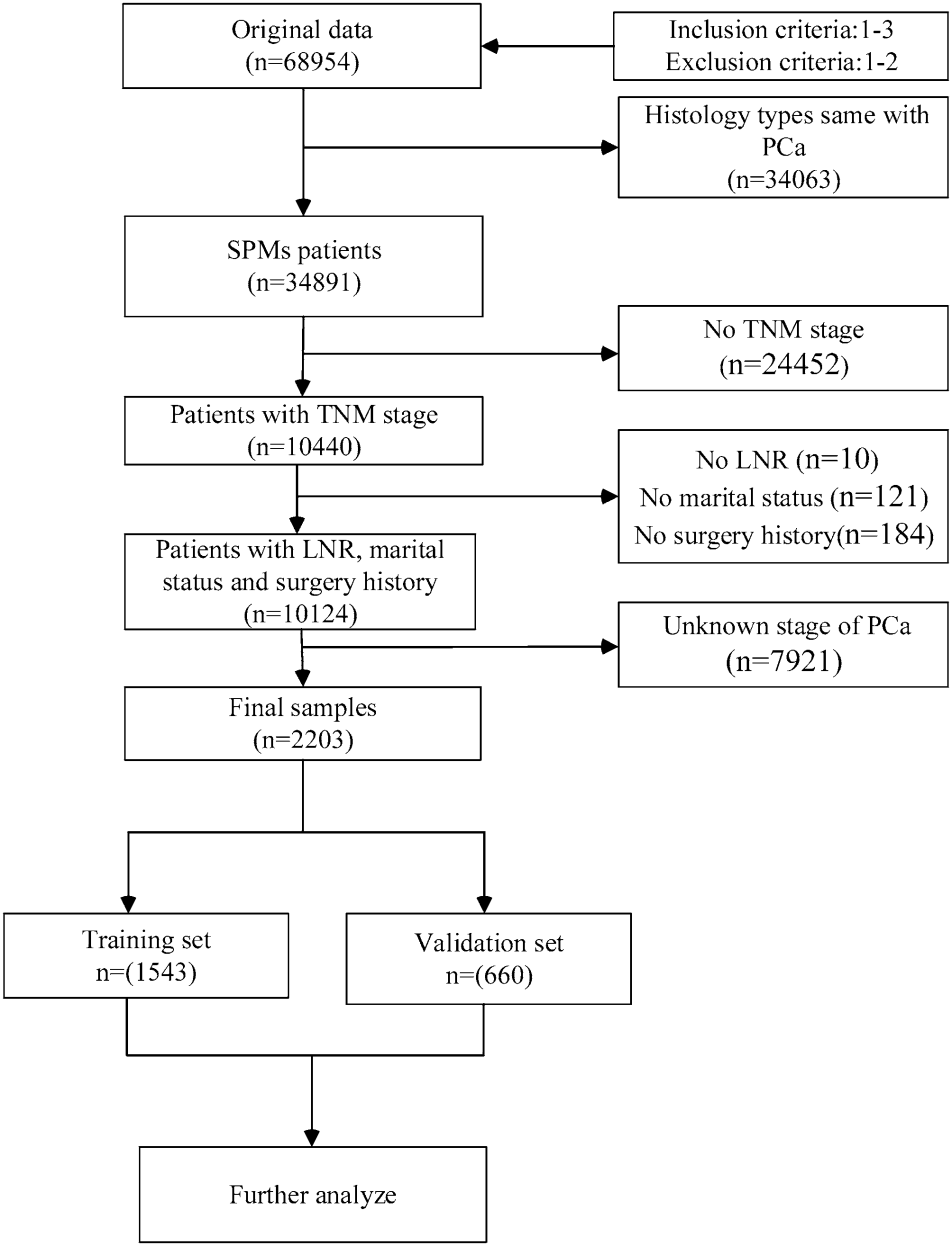
Study flowchart showing the process of constructing nomogram to predict the overall survival (OS) of second primary malignancies (SPMs) after prostate cancer (PCa).

The study cohort comprised patients with the following International Classification of Diseases for Oncology, Third Edition (ICD-O-3), morphology codes: 8000/3, 8010/3, 8140/3, 8255/3, 8480/3, 8481/3 and 8490/3, and the site codes: C61.9. The detailed flow chart for patient screening was presented in Fig. 1.

### Statistical analysis

To explore the association between clinicopathological variables and OS of SPM, we performed univariate and multivariate Cox proportional hazards regression analysis in the training set to identify the significant factors. Using these screened factors, we calculated the risk score of each patient according to the following formula: *risk score* = *β1* × *1* + *β2* × *2* + ⋯ + *βnXn* (β, regression coefficient; X, prognostic factor)^18^. According to the median score of the risk score, patients were divided into the high-risk group and low-risk group. Next, we chose factors with *p* value < 0.001 to develop a nomogram to predict the 3- and 5-year survival rates of SPM patients. To evaluate the prognostic ability of our model, we calculated the concordance index (C-index). Meanwhile, the receiver operating characteristic curve (ROC) was plotted and the area under the curve (AUC) was assessed. The calibration curves were drawn to estimate whether the actual result was consistent with the predicted probability. Each cohort was divided into three groups according to sample size. Bootstrapping with 1,000 resamples was used to evaluate discrimination and calibration. Kaplan–Meier curves were plotted and Log-rank analysis was applied to compare the OS on account of different prognostic factors.

All statistical analyses were performed in SPSS 24.0 (SPSS Inc., Chicago, IL, USA) or the R software (version 3.6.1; http://www.r-project.org/) using the following packages: ‘rms’, ‘survival’, and ‘survivalROC’. All tests were two-sided and *p* < 0.05 was considered statistically significant.

### Ethical statement

The authors are accountable for all aspects of the work in ensuring that questions related to the accuracy or integrity of any part of the work are appropriately investigated and resolved. Institutional review board approval was waived for this study because SEER database is a public anonymized database. The author Y Liu has gotten the access to the SEER database (accession number: 16704-Nov2018). The authors are accountable for all aspects of the work.

## Results

### Characteristics of SPM

**Figure 2:**
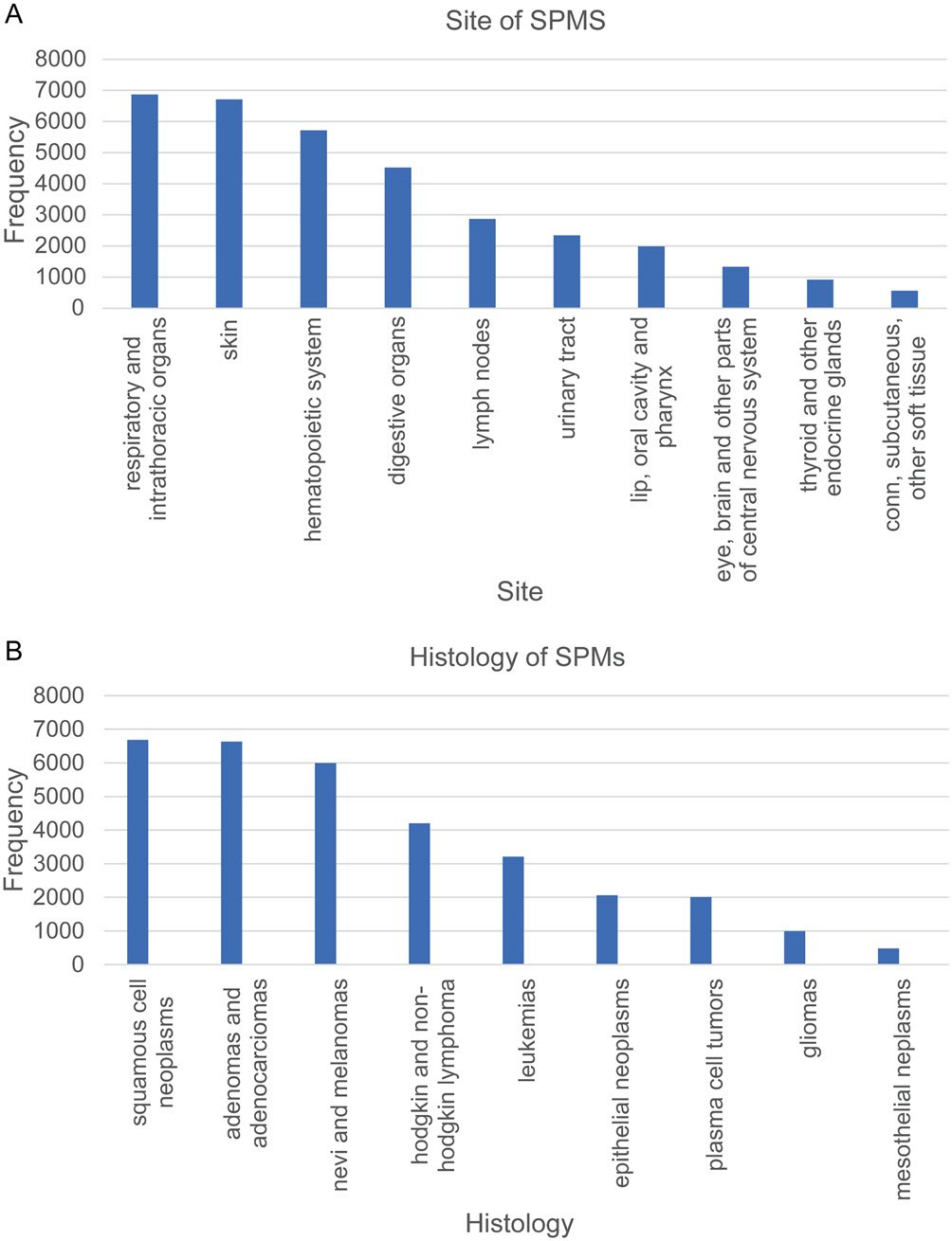
Features of second primary malignancies (SPMs) after prostate cancer (PCa). (**A**) Sites of SPMs that over than 1%, (**B**) Histology types of SPMs that more than 1%.

We downloaded 68,954 PCa patients diagnosed during 2000–2016 from the SEER database. In order to exclude the bias caused by PCa recurrence and metastasis, we ruled out cases with the same histological type as PCa. Cases with a latency period of less than 6 months between PCa and SPMs were also excluded. Finally, a total of 34,891 patients diagnosed with SPMs were identified. Using the SEER database, we found that 945,196 men were diagnosed during 2000–2016 and approximately 3.69% of PCa patients were subsequently diagnosed with SPMs in this period. We concluded that the median interval between diagnosis of PCa and SPM was 57.0 months and the median diagnosed age of SPM was 74.0 years. We listed the sites and histological types of SPM that exceeded 1% in Fig. 2A,B. The three most prevalent sites of SPM were respiratory and intrathoracic organs, skin, and hematopoietic system (Table 1). In addition, bronchial and lung cancers accounted for the majority of cancers in respiratory and intrathoracic organs (Table S1). As shown in Table 2, the top three histological types of SPMs were squamous cell carcinoma, adenoma and adenocarcinoma, nevi and melanoma.

**Table 1:** Site of SPMs after PCa.

Site of SPMs	N	N% (%)
All	34,891	100
Respiratory and intrathoracic organs	6866	19.68
Skin	6711	19.23
Hematopoietic system	5717	16.39
Digestive organs	4523	12.96
Lymph nodes	2872	8.23
Urinary tract	2343	6.72
Lip, oral cavity and pharynx	1985	5.69
Eye, brain and other parts of central nervous system	1331	3.81
Thyroid and other endocrine glands	917	2.63
Conn, subcutaneous, other soft tissue	563	1.61
Unknown primary site	348	1.00
Male genital organs	283	0.81
Bone, joints and articular cartilage	210	0.60
Retroperitoneum and peritoneum	125	0.36
Other and ill-defined sites	55	0.16
Breast	33	0.09
Peripheral nerves and autonomic nervous system	9	0.03

Abbreviations: SPMs: second primary malignancies; PCa: prostate cancer.

**Table 2:** Histology types of SPMs after PCa.

Histology type of SPMs	N	N% (%)
All	34,891	100
Squamous cell neoplasms	6686	19.16
Adenomas and adenocarcinomas	6634	19.01
Nevi and melanomas	5996	17.18
Hodgkin and non-Hodgkin lymphoma	4203	12.05
Leukemias	3214	9.21
Epithelial neoplasms	2064	5.92
Plasma cell tumors	2011	5.76
Gliomas	994	2.85
Mesothelial neoplasms	479	1.37
Myelodysplastic syndromes	278	0.80
Fibromatous neoplasms	259	0.74
Complex epithelial neoplasms	234	0.67
Complex mixed and stromal neoplasms	227	0.65
Lipomatous neoplasms	214	0.61
Soft tissue tumors and sarcomas	174	0.50
Transitional cell papillomas and carcinomas	145	0.42
Immunoproliferative diseases	136	0.39
Blood vessel tumors	132	0.38
Adnexal and skin appendage neoplasms	130	0.37
Ductal, lobular and medullary neoplasms	102	0.29
Chronic myeloproliferative disorders	96	0.28
Osseous and chondromatous neoplasms	95	0.27
Mucoepidermoid neoplasms	71	0.20
Thymic epithelial neoplasms	46	0.13
Germ cell neoplasms	46	0.13
Cystic, mucinous and serous neoplasms	29	0.08
Neoplasms	25	0.07
Meningiomas	25	0.07
Myomatous neoplasms	23	0.07
Nerve sheath tumors	20	0.06
Miscellaneous tumors	17	0.05
Other hematologic disorders	14	0.04
Paragangliomas and glomus tumors	12	0.03
Neuroepitheliomatous neoplasms	11	0.03
Myxomatous neoplasms	10	0.03
Neoplasms of histiocytes and accessory lymphoid cells	8	0.02
Synovial-like neoplasms	6	0.02
Odontogenic tumors	6	0.02
Basal cell neoplasms	5	0.01
Miscellaneous bone tumors	4	0.01
Mast cell tumors	4	0.01
Choriocarcinoma	2	0.01
Lymphatic vessel tumors	2	0.01
Giant cell tumors	2	0.01

Abbreviations: SPMs: second primary malignancies; PCa: prostate cancer.

### Baseline characteristics of patients

A total of 34,891 cases diagnosed with SPMs were identified from the original data downloaded from the SEER database. After excluding patients with unknown clinical information, 2203 cases were ultimately enrolled for further analysis. These cases were randomly divided into the training set (n = 1543) and the validation set (n = 660). There were no significant differences (*p* > 0.05) in the site of SPM, SPM histology, age, race, T stage, M stage, LNR, PCa surgery, PCa stage, and marital status (Table 3). The training set was used to construct nomogram and validate the model internally, while the validation set was used for external validation. In the entire cohort, we found that approximately 32.73% (n = 721) of SPM patients died after a median follow-up of 56 months.

**Table 3:** Characteristics of SPMs patients after PCa.

Characteristics	Training set (n = 1543)		Validation set (n = 660)		X^2^	*p*
	N	%	N	%		
**Site of SPMs**					4.91	0.423
Skin	439	28.5	188	28.5		
Bronchus and lung	298	19.3	111	16.8		
Renal	145	9.4	71	10.8		
Liver	85	5.5	48	7.3		
Thyroid gland	87	5.6	38	5.8		
Others	489	31.7	204	30.9		
**Histology of SPMs**					7.47	0.188
Squamous cell cancer	426	27.6	159	24.1		
Melanomas	413	26.8	173	26.2		
Papillary adenocarcinoma	118	7.6	62	9.4		
Hepatocellular carcinoma	75	4.9	44	6.7		
Renal cell carcinoma	79	5.1	29	4.4		
Others	432	28.0	193	29.2		
**Age**					3.40	0.334
<= 60	205	13.3	98	14.8		
61–70	589	38.2	268	40.6		
71–80	588	38.1	226	34.2		
> 80	161	10.4	68	10.3		
**Race**					0.71	0.703
White	1261	81.7	532	80.6		
Black	220	14.3	103	15.6		
Others	62	4.0	25	3.8		
**Stage_T**					1.22	0.875
Ta	71	4.6	27	4.1		
T1	673	43.6	301	45.6		
T2	344	22.3	149	22.6		
T3	279	18.1	111	16.8		
T4	176	11.4	72	10.9		
**Stage_N**					9.37	0.035
N0	1131	73.3	511	77.4		
N1	164	10.6	67	10.2		
N2	203	13.2	58	8.8		
N3	45	2.9	24	3.6		
**Stage_M**					1.52	0.218
M0	1359	88.1	594	90.0		
M1	184	11.9	66	10.0		
**LNR**					0.41	0.521
No	1214	78.7	528	80.0		
Yes	329	21.3	132	20.0		
**SPM surgical history**					0.79	0.374
Yes	521	33.8	210	31.8		
No	1022	66.2	450	68.2		
**Histology of PCa**					1.27	0.260
Other	13	0.8	9	1.4		
Ade	1530	99.2	651	98.6		
**PCa surgical history**					0.07	0.792
Yes	977	63.3	414	62.7		
No	566	36.7	246	37.3		
**PCa stage**					2.73	0.440
I	431	27.9	194	29.4		
II	905	58.7	373	56.5		
III	139	9.0	55	8.3		
IV	68	4.4	38	5.8		
**Marital status**						
Married	1030	66.8	426	64.5	0.13	0.936
Previously married	360	23.3	166	25.2		
Never married	153	9.9	68	10.3		

Abbreviations: SPMs: second primary malignancies; PCa: prostate cancer, LNR: lymph node removed.

### Prognostic factors for the overall survival of SPM

Intending to reveal the associated factors with the OS of SPM, we applied univariate and multivariate Cox regression analysis. The results were listed in Table 4. Univariate Cox regression analysis demonstrated that age (*p* < 0.001), race (*p* < 0.001), TNM stage (*p* < 0.001), LNR (*p* < 0.001), histology of SPM (*p* < 0.001), site of SPM (*p* < 0.001), marital status (*p* < 0.001), SPM surgical history (*p* < 0.001), PCa surgical history (*p* < 0.001), and PCa stage (*p* < 0.001) were associated with the OS of SPM. Next, using the factors identified by univariate Cox regression analysis, multivariate Cox regression analysis revealed that age (*p* < 0.001), TNM stage (*p* < 0.001), histology of SPM (*p* = 0.002), site of SPM (*p* < 0.001), marital status (*p* = 0.038), PCa surgical history (*p* < 0.001), and PCa stage (*p* < 0.001) were independent prognostic factors for the OS of SPM.

**Table 4:** Univariate and multivariate Cox analysis of SPMs patients after PCa in the training and validation set.

Characteristics	Univariate analysis			Multivariate analysis		
	HR	CI95	*p*	HR	CI95	*p*
**Site of SPM**			< 0.001			< 0.001
Skin	Reference			Reference		
Bronchus and lung	8.385	6.294–11.171	0.000	1.952	0.995–3.831	0.052
Renal	1.973	1.313–2.964	0.001	1.240	0.401–3.828	0.709
Liver	9.024	6.308–12.909	0.000	7.265	2.834–18.623	0.000
Thyroid gland	1.008	0.543–1.871	0.979	0.595	0.242–1.463	0.258
Others	2.671	1.990–3.585	0.000	0.978	0.513–1.866	0.947
**Histology of SPM**			< 0.001			0.002
Squamous cell neoplasms	Reference			Reference		
Melanomas	0.199	0.147–0.269	0.000	0.549	0.278–1.085	0.085
Papillomas	0.292	0.186–0.458	0.000	0.921	0.412–2.058	0.841
Hepatocellular carcinoma	1.742	1.277–2.375	0.000	0.576	0.268–1.242	0.159
Adenocarcinomas	0.553	0.362–0.845	0.006	1.072	0.382–3.011	0.895
Others	0.815	0.666–0.997	0.047	1.364	1.084–1.717	0.008
**Age**			< 0.001			< 0.001
<= 60	Reference			Reference		
61–70	1.211	0.885–1.657	0.231	1.130	0.818–1.561	0.458
71–80	1.861	1.373–2.521	0.000	1.692	1.224–2.340	0.001
> 80	2.465	1.734–3.504	0.000	2.354	1.619–3.424	0.000
**Race**			< 0.001			0.638
White	Reference			Reference		
Black	1.605	1.287–2.001	0.000	1.040	0.821–1.317	0.747
Other	1.003	0.640–1.571	0.991	0.817	0.515–1.297	0.392
**Stage_T**			< 0.001			< 0.001
T1	Reference			Reference		
Ta	1.381	0.873–2.186	0.167	1.443	0.878–2.372	0.148
T2	2.108	1.665–2.669	0.000	1.334	1.034–1.721	0.026
T3	2.930	2.309–3.719	0.000	1.626	1.242–2.127	0.000
T4	3.825	2.952–4.956	0.000	1.871	1.386–2.526	0.000
**Stage_N**			< 0.001			< 0.001
N0	Reference			Reference		
N1	2.710	2.128–3.450	0.000	2.028	1.552–2.65	0.000
N2	3.516	2.838–4.357	0.000	1.493	1.152–1.934	0.002
N3	4.299	2.913–6.346	0.000	1.739	1.128–2.679	0.012
**Stage_M**			< 0.001			< 0.001
M0	Reference			Reference		
M1	7.664	6.305–9.315	0.000	2.893	2.292–3.651	0.000
**LNR**			< 0.001			0.088
No	Reference			Reference		
Yes	0.600	0.474–0.760	0.000	0.757	0.555–1.033	0.079
**SPM surgical history**			< 0.001			< 0.001
Yes	Reference			Reference		
No	0.198	0.165–0.236	0.000	0.597	0.452–0.790	0.000
**Histology of PCa**			0.134			
Other	Reference			Reference		
Adenocarcinomas	0.538	0.240–1.203	0.131			
**PCa surgical history**			< 0.001			0.450
Yes	Reference			Reference		
No	0.600	0.495–0.726	0.000	0.930	0.751–1.151	0.503
**PCa Stage**			< 0.001			< 0.001
I	Reference			Reference		
II	1.143	0.934–1.399	0.193	1.046	0.85–1.288	0.668
III	0.745	0.512–1.086	0.126	0.856	0.571–1.281	0.449
IV	2.411	1.704–3.410	0.000	2.419	1.684–3.476	0.000
**Marital status**			< 0.001			0.038
Married	Reference			Reference		
Previously married	1.391	1.141–1.696	0.001	1.120	0.914–1.372	0.275
Never married	1.477	1.126–1.939	0.005	1.469	1.104–1.955	0.008

Abbreviations: SPM: second primary malignancy; PCa: prostate cancer, LNR: lymph node removed.

### Kaplan–Meier analysis for prognostic factors

**Figure 3:**
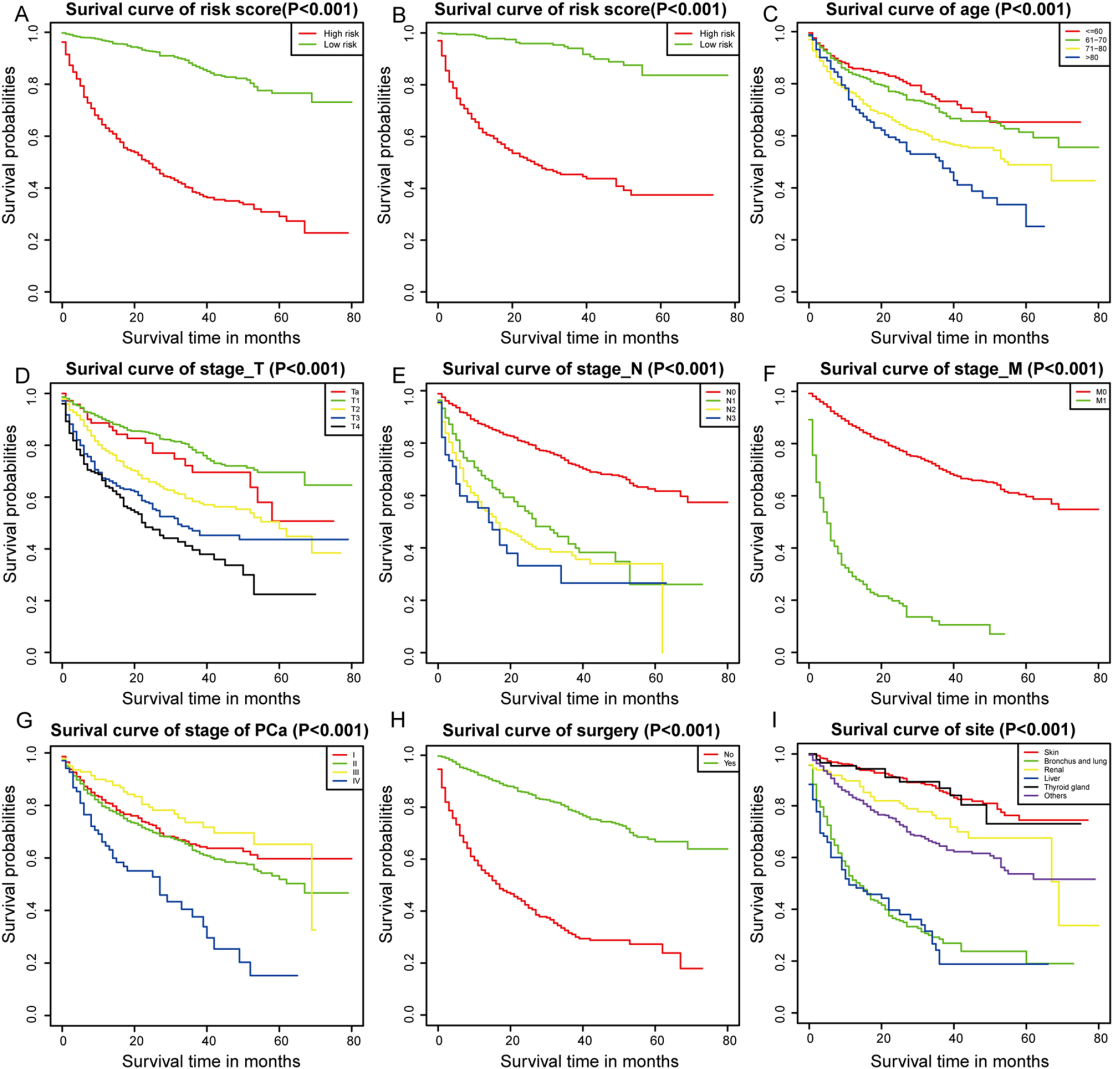
Kaplan–Meier analysis for overall survival (OS) of second primary malignancy (SPM) after prostate cancer (PCa) based on risk score in the training set (*p* < 0.001) (**A**) and the validation set (*p* < 0.001) (**B**), age (*p* < 0.001) (**C**), stage T (*p* < 0.001) (**D**), stage N (*p* < 0.001) (**E**), stage M (*p* < 0.001) (**F**), PCa stage (*p* < 0.001) (**G**), SPM surgery history (*p* < 0.001) (**H**), and site of SPMs (*p* < 0.001) (**I**).

We first calculated the risk score of each case according to the following formula: *risk score* = *β1* × *1* + *β2* × *2* + ⋯ + *βnXn* (β, regression coefficient; X, prognostic factor). Then, we divided samples into the high-risk group and low-risk group based on the media risk score. Kaplan–Meier (K–M) analysis showed significant differences in the prognosis between these two groups in the training set and validation set (Fig. 3A,B) and patients with high risks tended to have worse survival than those with low risk (*p* < 0.001). Significant differences were also observed in site of SPM (*p* < 0.001), age, TNM stage (*p* < 0.001), SPM surgery history (*p* < 0.001), and PCa stage (*p* < 0.001). Patients with higher age, TNM stage, PCa stage had better survival (Fig. 3C,G). Also, patients who received surgery for SPM tended to have increased survival (Fig. 3H). SPM of skin had significantly better survival than other kinds of SPM (Fig. 3I).

### Construction and validation of OS nomogram

**Figure 4:**
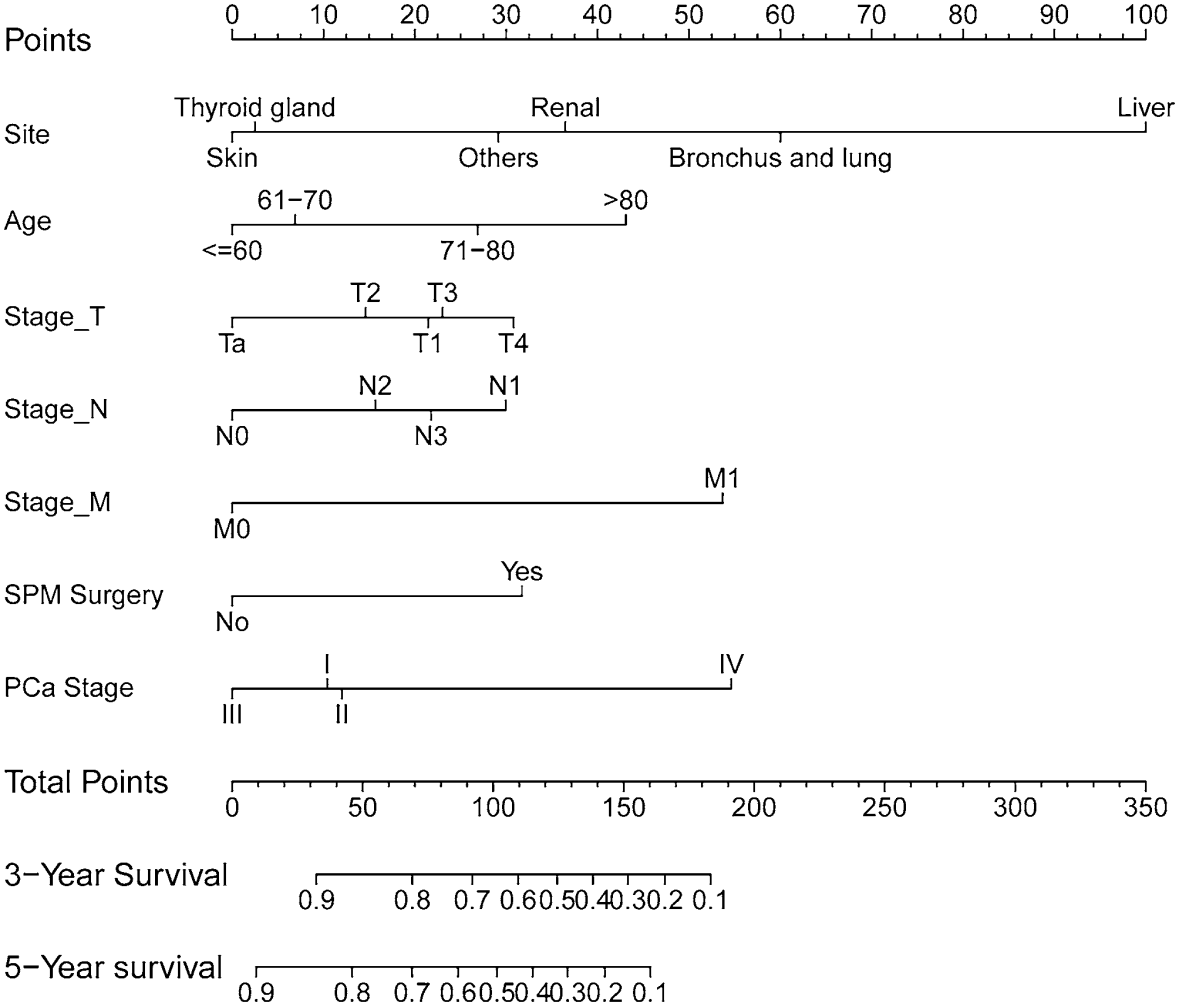
Nomogram to predict 3- and 5-year survival for second primary malignancy (SPM) patients.

**Figure 5:**
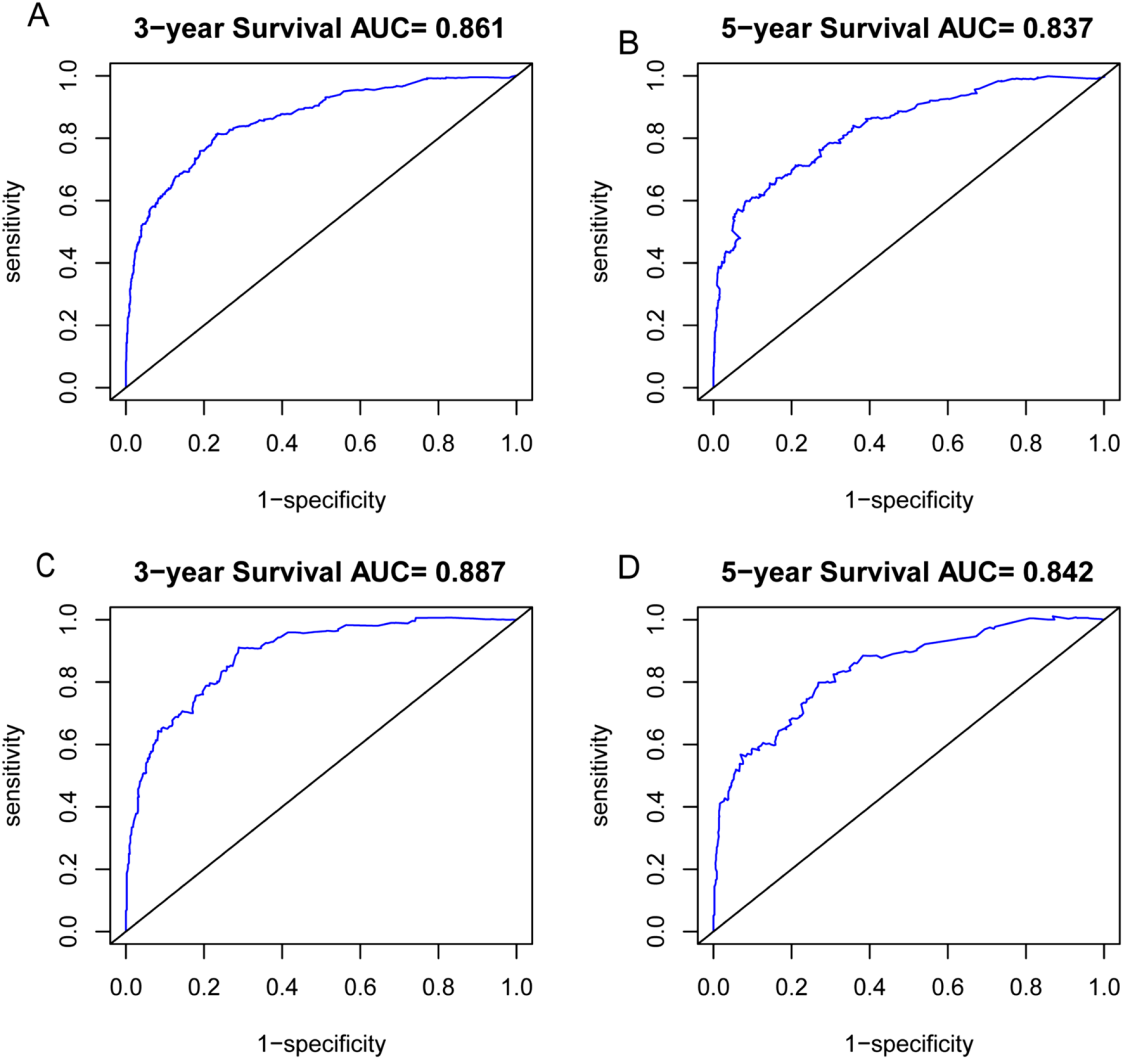
Receiver operating characteristic (ROC) analysis to assess 3-year (**A**) and 5-year (**B**) survival for second primary malignancy (SPM) patients in the training set; The ROC curve to assess 3-year (**C**) and 5-year (**D**) survival in the validation set.

According to the results of univariate and multivariate Cox analysis, we chose the factors with *p *value < 0.001 to establish a nomogram to predict the 3-year and 5-year survival rate (Fig. 4). Seven clinical indicators, including site of SPM, age, TNM stage, SPM surgical history, and PCa stage were enrolled in our nomogram. In order to evaluate the discriminative ability of the nomogram constructed by us, we calculated the C-index in the training set (0.824, 95% CI: 0.806–0.842) and validation set (0.862, 95% CI: 0.840–0.884). The ROC was plotted and AUC was analyzed for both the training set and validation set (Fig. 5A–D). The AUCs in the training set used for 3-year and 5-year OS predication were 0.861 and 0.837, respectively. In the validation set, values of AUCs for 3-year and 5-year OS predication were 0.887 and 0.842. Both the C-index and the ROC indicated that the nomogram we constructed performed well in predicting the OS of SPM.

**Figure 6:**
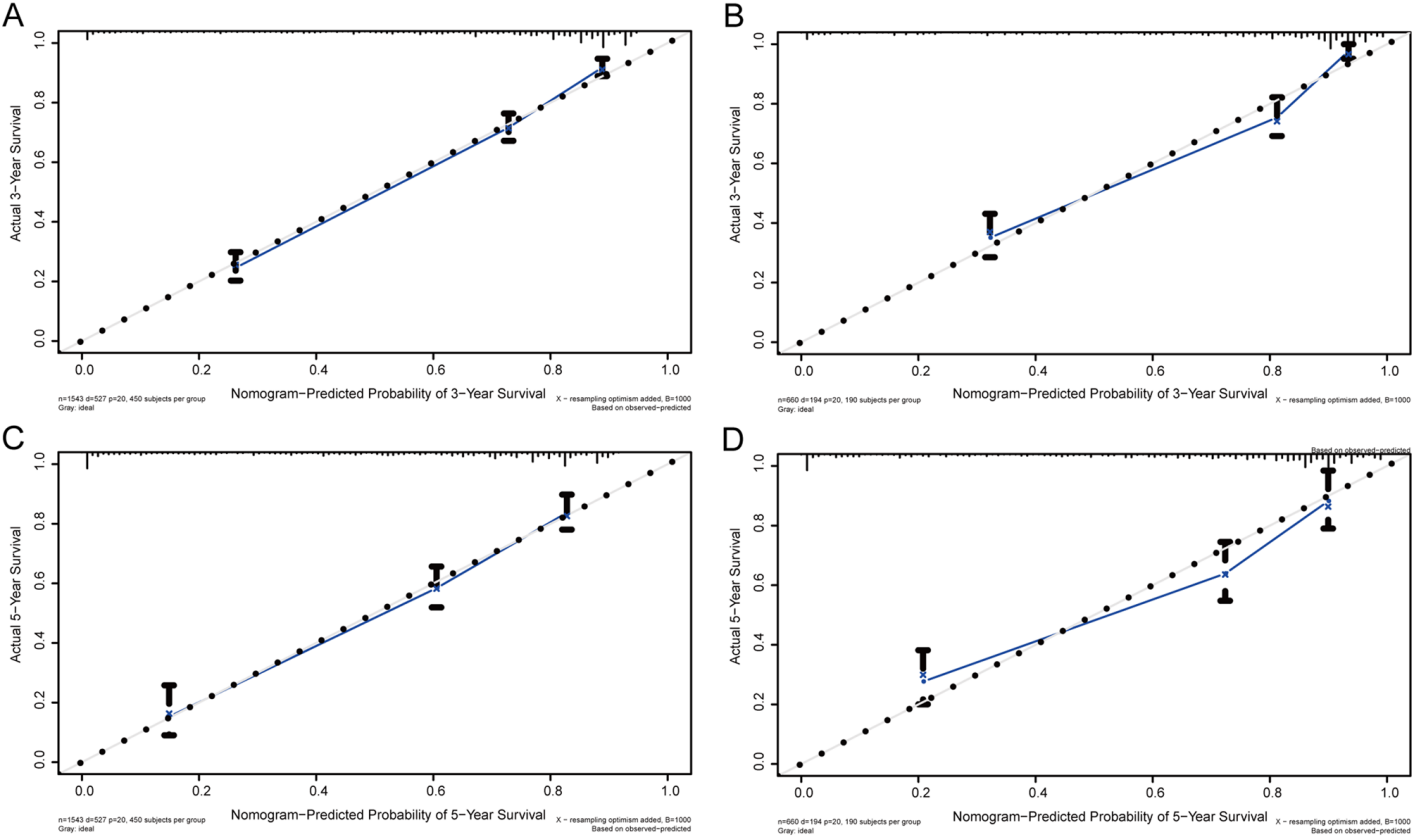
The calibration curve to evaluate the 3-year (**A**) and 5-year (**B**) survival for second primary malignancy (SPM) patients in training set; The calibration curve to evaluate the 3-year (**C**) and 5-year (**D**) survival for SPMs patients in the validation set. Nomogram-predicted overall survival (OS) is plotted on the x-axis; actual OS is plotted on the y-axis. The imaginary line indicates a perfect calibration model in which the predicted probabilities are identical to the actual survival outcomes.

In order to evaluate the accuracy of our model, we also used the calibration plots to judge the consistency of our predictions with actual outcomes (Fig. 6A–D). Figures presented an acceptable agreement in the training cohort and an excellent agreement in SEER validation cohort between the nomogram predictions and actual observations for 3-year and 5-year OS.

## Discussions

As the most common cancer among males, the survival time of PCa patients has been significantly extended due to early detection and effective therapeutic strategies. PSA screening is helpful for early diagnosis, and can significantly reduce the mortality rate of PCa^20^. For decades, ADT through surgical or medical castration has been part of the standard treatment for PCa. Newly launched second-generation androgen receptor (AR) inhibitors for castration-resistant prostate cancer (CRPC), such as enzalutamide, also show significant capacities of improving the prognosis of PCa^21^. Recently, the use of ADT in combination with second-generation AR targeting agents or chemotherapy has significantly prolonged the longevity of metastatic hormone sensitive prostate cancer (mCRPC) patients. The addition of abiraterone acetate to ADT has shown a survival advantage compared to using ADT alone^22,23^. Two clinical trials have shown that, compared with alone, ADT plus docetaxel can improve the survival rate for adequately fit men^24,25^. All these advanced treatments have together contributed to the prolonged survival of PCa patients. Previous studies have shown that patients with in situ melanoma have an increased risk of developing PCa^26^ and young men among colorectal cancer survivors have an excessively high risk of developing SPMs^27^. These evidences indicate that cancer patients had chances of developing SPMs. Studies in South Korea and Taiwan show that compared to the general population, PCa patients have a lower risk of SPMs, but once they got SPMs, the survival time of PCa patients will be greatly shortened^28,29^. For the reason of better insight into SPMs after PCa, we investigated the characteristics of SPM following PCa, and constructed a model based on clinicopathologic characteristics to predict the prognosis of SPM following PCa.

As a result of the extended survival period of PCa patients, recurrence, metastasis, and SPMs are expected to increase. In clinical practice, SPMs or multiple primary malignancies are very frequently indistinct from the metastasis of initial malignancy, leading to misdiagnosis and improper treatment of patients. In contrast to multiple primary malignancies, SPMs can affect the same organ but are anatomically distinct from the primary tumor, and represent neither a metastatic nor recurrent tumor from the initial malignancy. Via a strict screening process, we distinguished between SPMs from multiple primary malignancies, metastasis, and recurrence. After accurate identification, 3.69% of PCa patients were diagnosed with SPMs, which was much lower than previous 11.3%^8^. Compared with previous studies, our investigation enrolled a much larger population, containing 945,196 SPM samples. Our study showed that the three most prevalent sites of SPM were skin, hematopoietic system, bronchus and lung. Similar to our results, previous studies in Sweden reported that the most common SPMs were colorectal cancer, skin cancer, bladder cancer, lung cancer, melanoma, and non-Hodgkin lymphoma^8^. Another study also showed that the most common cancers of SPMs after PCa were lung and colon cancer^30^. In addition to these three most prevalent sites, a significant increase of SPM in the urinary tract was also observed in our study. It has been reported that there is an increased risk of developing SPMs in the bladder^13,29^. Shared etiology of the urinary system, such as common carcinogenic pathways, chronic inflammatory stimulation, and genetic mutations^31^, may be the reasons for this trend. The top three histological types of SPM were squamous cell carcinoma, adenoma and adenocarcinoma, nevi and melanoma, consistent with the histology of epidemic sites. These results indicated that these prone sites should be cautiously monitored.

Former researches have established nomograms to predict the probability of getting SPMs, including lung cancer survivors^32,33^, esophageal adenocarcinoma and squamous cell carcinoma patients^34^. However, as far as we know, there is no literature on the prognosis across the spectrum of PCa patients subsequently diagnosed with SPMs. In order to explore the outcome of SPM following PCa, we identified 7 parameters, including the site of SPM, age, TNM stage, SPM surgical history, and PCa stage, to predict the 3-year and 5-year OS of SPM patients. According to our assessment, our model performed well in predicting the outcomes of SPM patients. Of all these factors, surgical history of PCa and histological type of PCa presented a weak correlation with the outcome of SPM, which might suggest that SPM mainly accounted for the death of SPM following PCa. Besides, researchers also found that most causes of death were caused by SPM not PCa^11,35^. PCa stage was enrolled in our nomogram, and was used to construct a predicting model for metastatic PCa together with TNM stage^36,37^. We could conclude that PCa still had its impact on the OS of SPM.

However, we did not investigate the relationship between RT and SPM. According to earlier reports, PCa patients receiving RT have a higher risk of getting SPMs^38–40^. A meta-analysis also reveals that PCa patients receiving RT had an increasing risk of developing SPM of the bladder, colon, and rectum^41^. Some studies have shown that there is no difference in the incidence of SPM among patients receiving RT or other therapies^13,42^. The role of RT in the initiation of SPM still needs more exploration and the effect of RT on the survival of PCa patients remains unclear. Gene is another important internal factor of tumorigenesis of SPM, but the genotype–phenotype correlation of SPMs is still unclear. A significantly increased risk of SPM has been observed in survivors of hereditary retinoblastoma with high RB1 mutations^43^. P53 gene whose polymorphisms are associated with an increased risk of SPM is another gene extensively researched^44–46^. On the contrary, Anette. E et.al believed the correlation between P53 mutation and the incidence of SPM was doubtful^47^. Only limited evidence about the SPM genotype were explored and more studies are needed to explain the relationship between SPMs and gene mutations. Some other factors, such as smoking and obesity, were not investigated due to the nature of the SEER database. We are trying to explore the association between cancer-specific survival and clinicopathologic characteristics, but the causes of tumor death in many patients are still vague. Despite these limitations, our study still has its implications for PCa survivors.

In conclusion, we described the general characteristics of SPM following PCa and identified 7 clinical indicators to build a nomogram to predict the survival of SPM. The model we constructed performed well in assessing the prognosis of SPM but its actual efficiency should be evaluated with more large-scale researches. In addition, more studies should focus on the initiation, development, and prognosis of SPM.

## Supplementary Information

Supplementary Table.
