# Supplementary material for: Clinical characteristics and overall survival nomogram of second primary malignancies after prostate cancer, a SEER population-based study
Source: Sci Rep. 2021 Jan 14;11:1293. doi: 10.1038/s41598-020-80534-4 (PMC7809269; doi:10.1038/s41598-020-80534-4)
Supplement: Supplementary file 1 — Supplementary Table. [file 41598_2020_80534_MOESM1_ESM.docx]

Table S1：

Site of SPMs after PCa

| **Site of SPMs** | **Detail Site of SPMs** | **N** | **Total (%)** |
| --- | --- | --- | --- |
| lip, oral cavity and pharynx | lip | 161 | 1985 (5.69%) |
|  | base of tongue | 394 |  |
|  | other and unspecified parts of tongue | 176 |  |
|  | gum | 41 |  |
|  | mouth | 90 |  |
|  | palate | 75 |  |
|  | other and unspecified parts of mouth | 86 |  |
|  | parotid gland | 193 |  |
|  | other and unspecified major salivary glands | 43 |  |
|  | tonsillar | 334 |  |
|  | oropharynx | 84 |  |
|  | nasopharynx | 106 |  |
|  | pyriform sinus | 94 |  |
|  | hypopharynx | 67 |  |
|  | other and ill-defined sites in lip, cavity and pharynx | 41 |  |
| digestive organs | esophagus | 432 | 4523 (12.96%) |
|  | stomach | 625 |  |
|  | small intestine | 158 |  |
|  | colon | 1027 |  |
|  | rectosigmoid junction | 76 |  |
|  | rectum | 337 |  |
|  | anus and anal canal | 157 |  |
|  | liver and intrahepatic bile duct | 1407 |  |
|  | gallbladder | 30 |  |
|  | other and unspecified parts of biliary tract | 154 |  |
|  | pancreas | 100 |  |
|  | other and ill-defined digestive organs | 20 |  |
| respiratory and intrathoracic organs | nasal cavity and middle ear | 87 | 6866 (19.68%) |
|  | accessory sinuses | 57 |  |
|  | larynx | 839 |  |
|  | trachea | 9 |  |
|  | bronchus and lung | 5369 |  |
|  | thymus | 57 |  |
|  | heart, mediastinum and pleura | 448 |  |
| bone, joints and articular cartilage | bone, joints and articular cartilage | 45 | 210 (0.60%) |
|  | bone, joints and articular cartilage of other and unspecified sites | 165 |  |
| hematopoietic system | hematopoietic system | 5717 | 5717 (16.39%) |
| skin | skin | 6711 | 6711 (19.23%) |
| peripheral nerves and autonomic nervous system | peripheral nerves and autonomic nervous system | 9 | 9 (0.03%) |
| retroperitoneum and peritoneum | retroperitoneum and peritoneum | 125 | 125 (0.36%) |
| conn, subcutaneous, other soft tissue | conn, subcutaneous, other soft tissue | 563 | 563 (1.61%) |
| breast | breast | 33 | 33 (0.09%) |
| male genital organs | penis | 127 | 283 (0.81%) |
|  | prostate gland | 15 |  |
|  | testis | 98 |  |
|  | other and unspecified male genital organs | 43 |  |
| urinary tract | kidney | 2023 | 2343 (6.72%) |
|  | renal pelvis | 44 |  |
|  | ureter | 38 |  |
|  | bladder | 197 |  |
|  | other and unspecified urinary organs | 41 |  |
| eye, brain and other parts of central nervous system | eye and adnexa | 195 | 1331 (3.81%) |
|  | meninges | 24 |  |
|  | brain | 1074 |  |
|  | spinal cord, cranial, nerves and other parts of central nervous system | 38 |  |
| thyroid and other endocrine glands | thyroid gland | 882 | 917  (2.63%) |
|  | adrenal gland | 24 |  |
|  | other endocrine glands and related structures | 11 |  |
| other and ill-defined sites | other and ill-defined sites | 55 | 55 (0.16%) |
| lymph nodes | lymph nodes | 2872 | 2872  (8.23%) |
| unknown primary site | unknown primary site | 348 | 348 (1.00%) |
| All sites | All sites | 34891 | 34891  (100%) |

Abbreviation: SPMs: second primary malignancies; PCa: prostate cancer
